# The sFlt-1/PlGF Ratio Trend Is Useful in Predicting Preeclampsia Severity in Hyperreactio Luteinalis Complicated with Preeclampsia

**DOI:** 10.1155/2023/7352947

**Published:** 2023-09-19

**Authors:** Risa Miyatake, Tatsuya Fujii, Keiichi Kumasawa, Mari Ichinose, Masatake Toshimitsu, Seisuke Sayama, Takahiro Seyama, Takayuki Iriyama, Takeshi Nagamatsu, Yutaka Osuga

**Affiliations:** ^1^Department of Obstetrics and Gynecology, Faculty of Medicine, The University of Tokyo, 113-8655, Japan; ^2^Department of Obstetrics and Gynecology, National Center for Child Health and Development, 157-8535, Japan; ^3^Department of Obstetrics and Gynecology, International University of Health and Welfare, 286-0124, Japan

## Abstract

Hyperreactio luteinalis (HL) is a rare condition that presents as bilateral ovarian enlargement during pregnancy. Typically, it is thought to be caused by increased production of human chorionic gonadotropin (hCG) associated with gestational trophoblastic diseases or multiple pregnancies. The prognosis is relatively good, with many cases resulting in term birth. However, some obstetric complications, such as preeclampsia (PE) and preterm births, have been reported. We present a serious case of HL with subsequent PE that resulted in preterm delivery at 31 weeks of gestation. The soluble fms-like tyrosine kinase-1 (sFlt-1)/placental growth factor (PlGF) ratio was very high at the onset of PE at 24 weeks of gestation, followed by a modest decline, which then increased in proportion to the exacerbation of symptoms. Since HL cases have also been reported to be associated with PE, repeated measurement of the sFlt-1/PlGF ratio proved useful for better pregnancy management.

## 1. Introduction

Hyperreactio luteinalis (HL) is a rare benign condition during pregnancy characterized by multicystic bilateral ovarian enlargement, increased serum human chorionic gonadotropin (hCG) levels, and spontaneous regression after delivery.

In the analysis of 96 HL cases, 24% had preeclampsia (PE), and 12% had fetal growth restriction (FGR) [1]. Only four cases were affected by preterm births due to worsening PE, as in this case [2, 3, 4, 5].

## 2. Case Report

**Figure 1:**
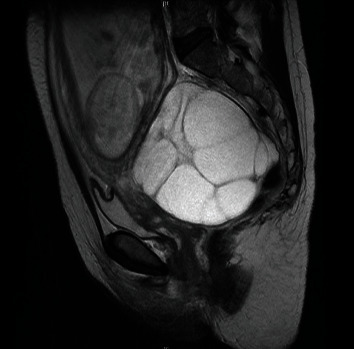
T2-weighted images show bilateral ovarian enlargement, which looks like HL. MRI of the patient's pelvic at the gestational age of 19 weeks.

**Figure 2:**
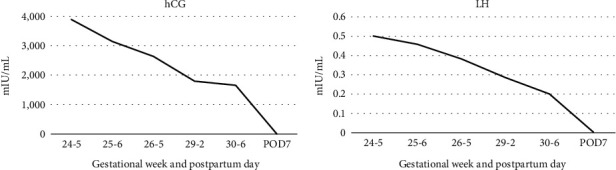
The transition of the hormone levels, hCG and LH from 24 weeks of gestation to postpartum period. The trend of hCG and LH.

**Figure 3:**
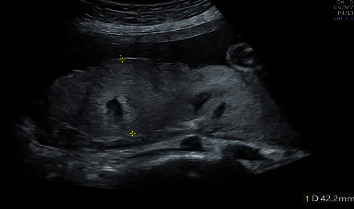
The thickness was 42.2 mm. Placenta by ultrasonography at the gestational age of 25 weeks.

**Figure 4:**
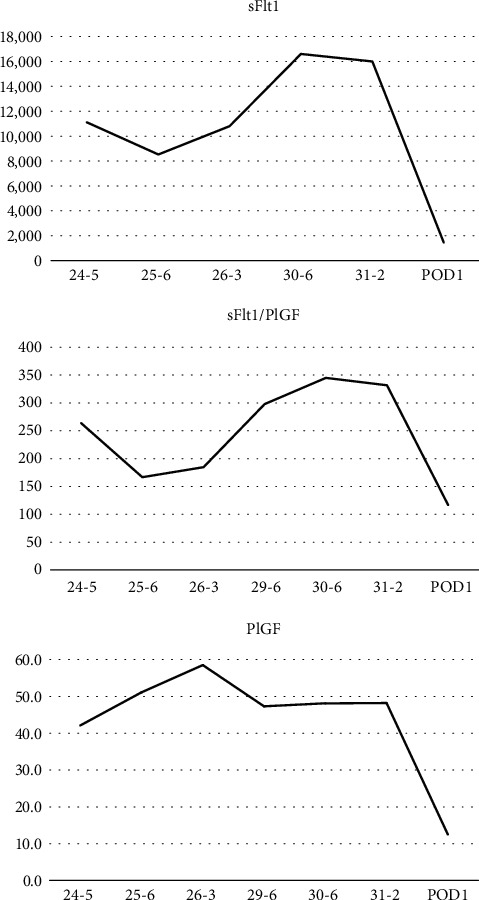
The transition from 24 weeks gestation to postpartum period. The sFlt-1 level was very high at 24 weeks gestation and once gradually decreased but rapidly increased again. Immediately before delivery, the sFlt-1 level was maximum. All marker decreases rapidly after birth.

A 37-year-old G1P0 woman conceived naturally without any fertility drugs and was referred to our hospital for delivery at 18 weeks and 5 days of gestation. She had been taking levothyroxine for hypothyroidism since early pregnancy. The thyroid stimulating hormone (TSH) level was 1.22 mIU/ml, and the fT4 level was 1.35 ng/dl at 15 weeks of gestation. On the first visit to our hospital, a transvaginal ultrasound confirmed bilateral multicystic ovarian enlargement of up to approximately 9 cm along the major axis. There were no substantial areas, and the possibility of malignancy was considered low; therefore, the patient was carefully monitored. In the fetus, no obvious morphological abnormalities were observed; however, FGR, with an estimated weight of 162 g (-2.3SD) was confirmed. At 19 weeks and 2 days of gestation, she visited our hospital complaining of mild lower abdominal pain. Magnetic resonance imaging revealed no ascites, malignant findings, or torsion of the adnexal pedicle (Figure 1). Subsequently, her lower abdominal pain resolved spontaneously. At 24 weeks and 5 days of pregnancy, she was diagnosed with PE due to hypertension, with a systolic blood pressure of 152 mmHg and proteinuria which was an evaluated ratio of Tp/Cre during a prenatal check-up, and was hospitalized. The fetal weight was estimated to be 549 g (-2.3 SD) and was still in the FGR. In addition, a serum hCG level of 388,662 mIU/ml was detected, and the luteinizing hormone (LH) level was 50.1 IU/L (Figure 2). The bilateral ovarian enlargement remained unchanged. She was diagnosed with HL based on these findings. Abdominal ultrasonography revealed a slightly swollen placenta (Figure 3). Serum soluble fms-like tyrosine kinase-1 (sFlt-1) and placental growth factor (PlGF) levels were measured by means of electrochemiluminescence immunoassay platform (cobas e analyzers, Roche Diagnostics) and were 11,000 pg/mL and 42.1 pg/mL, respectively; the sFlt-1/PlGF ratio, which is associated with prediction of PE development, was remarkably high at 263.7 (Figure 4).

For approximately 4 weeks after admission, systolic blood pressure remained within 130–150 mmHg without medication, and no other preeclamptic findings worsened. The hCG levels decreased after that. A mild decrease in sFlt-1 levels and no remarkable change in PlGF levels were observed during the 4 weeks.

Around 29 weeks gestation, PE gradually worsened, and blood pressure reached 160/88 mmHg. In addition, a rapid increase in the sFlt-1/PlGF ratio was observed, which reached 345.1 at 30 weeks and 6 days of gestation in conjunction with the severity of PE. Therefore, maternal prophylactic betamethasone administration was performed for fetal lung maturation. Emergency cesarean section was performed at 31 weeks and 2 days of gestation due to worsened PE with uncontrolled blood pressure and rapidly decreasing platelet counts down to 10.8 × 10^4^/*μ*L (Table 1).

**Figure 5:**
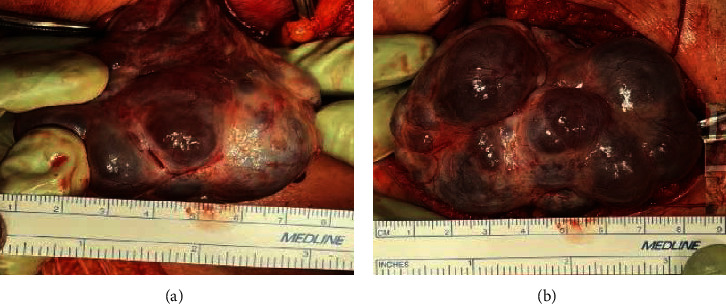
Long axis length of (a, b) ovary: 5 cm/9 cm.

A 1,099 g baby girl was delivered with an Apgar score of 5/8 and umbilical artery blood gas pH of 7.32. The placenta weighed 359 g and showed no abnormalities. Histological examination revealed no vascular infarctions or ischemic changes. Intraoperative findings showed that the bilateral ovaries were polycystic and enlarged to 8 cm in diameter (Figure 5). The serum hCG level decreased markedly to 691 mIU/ml 7 days after delivery. Ultrasonographic findings of the enlarged polycystic ovaries improved. Postpartum progress was good, and the patient was discharged on the eighth postoperative day. Her blood pressure had normalized at the 1-month postpartum check-up, and there was no evidence of bilateral ovarian enlargement. The baby was managed in the neonatal intensive care unit after birth and discharged at 76 days of age with no apparent adverse events. At the 4-month check-up, growth and development were equivalent to age.

Examination of the placental chromosome revealed a balanced reciprocal translocation of the short arm of chromosome 2 and the long arm of chromosome 18. In addition, a chromosomal examination of the patient and her husband revealed a balanced translocation of paternal origin.

## 3. Discussion

We encountered a case of HL complicated by PE. An emergency cesarean section was performed at 31 weeks of gestation due to exacerbating PE. This is the first report of sequential examinations of the sFlt-1/PlGF ratio for HL-complicated pregnancies from the onset of PE to the postpartum period.

Currently, the pathogenesis of HL is not fully understood. However, it has been reported that HL is caused by increased hCG production and sensitivity to gonadotropins associated with trophoblastic disease and twin pregnancies, resulting in nonneoplastic multifocal enlargement of the ovaries during pregnancy [1]. In this case, LH and hCG levels were elevated, even though the patient had a singleton pregnancy without trophoblastic disease.

Masuyama et al. reported that the sFlt-1/PlGF ratio increased from 14.9 to 161.1 from 18 to 31 weeks of gestation in PE patients with HL [3]. Our study is the first to observe changes in the sFlt-1/PlGF ratio throughout pregnancy in patients with HL. In our case, the sFlt-1/PlGF ratio was even higher: 263.7 at the onset of PE at 24 weeks gestation and 345.1 at 30 weeks gestation. After 24 weeks of gestation, the sFlt-1/PlGF ratio decreased temporarily, along with the patient's stable condition. After that, the sFlt-1/PlGF ratio rapidly increased as PE worsened at around 29 weeks of gestation. sFlt-1/PlGF is widely adopted as a predictive marker for PE and often shows high values in onset cases. Moreover, it has been reported that the higher the value, the more severe the disease [6]. There are also reports showing exacerbation of PE with a steep rise in sFlt-1/PlGF [7].

In the present case, the sFlt-1/PlGF ratio was already extremely high at 24 weeks of gestation when the patient developed PE; however, without preeclamptic exacerbation, her delivery was extended to 31 weeks of gestation. During the course, sFlt-1/PlGF slightly improved; however, PE symptoms were exacerbated when it was extremely elevated again.

In HL cases with PE and FGR, repeated measurement of the sFlt-1/PlGF ratio may be useful for better pregnancy management.

## Figures and Tables

**Table 1:** Physical findings and laboratory data from admission.

	Gestational age						
	24w5d	25w3d	29w2d	30w6d	31w2d	POD4^∗^^1^	POD7
Blood pressure (mmHg)	152/77	136/75	160/86	137/88	155/89	153/90	126/70
Weights (kg)	56.6	57.1	58.8	58.9	58.3	54.1	53.1
EFW^∗^^2^ (g)	549	644	1079	1184	1099	—	—
AST^∗^^3^ (U/L)	36	28	18	21	21	112	28
ALT^∗^^4^ (U/L)	54	36	10	18	14	94	52
Platelets (×10,000/*μ*l)	16.3	15.5	13.9	12.3	10.8	14.4	21.3
Tp/Cre	—	—	117.6	330.4	481	614	321.4

^∗^^1^POD: postoperative day; ^∗^^2^EFW: estimated fetal weight; ^∗^^3^AST: aspartate aminotransferase; ^∗^^4^ALT: alanine aminotransferase.

## Data Availability

Clinical data is restricted to associated members.
